# PreImplantation Factor in endometriosis: A potential role in inducing immune privilege for ectopic endometrium

**DOI:** 10.1371/journal.pone.0184399

**Published:** 2017-09-13

**Authors:** Marco Sbracia, Brett McKinnon, Fabio Scarpellini, Daniela Marconi, Gabriele Rossi, Cedric Simmilion, Michael D. Mueller, Eytan R. Barnea, Martin Mueller

**Affiliations:** 1 Hungaria Center for Endocrinology and Reproductive Medicine, Rome, Italy; 2 Department of Clinical Research, University of Bern, Bern, Switzerland; 3 Department of Obstetrics and Gynecology, Università degli Studi di Roma Tor Vergata, Rome, Italy; 4 Department of Obstetrics and Gynecology, University Hospital Bern, Bern, Switzerland; 5 SIEP- The Society for the Investigation of Early Pregnancy, Cherry Hill, NJ, United States of America; 6 Department of Research and Development, BioIncept LLC, Cherry Hill, NJ, United States of America; 7 Department of Obstetrics, Gynecology, and Reproductive Sciences, Yale University School of Medicine, New Haven, CT, United States of America; Universite Paris-Sud, FRANCE

## Abstract

Endometriosis is a chronic inflammatory condition characterised by the growth of endometrial epithelial and stromal cells outside the uterine cavity. In addition to Sampson’s theory of retrograde menstruation, endometriosis pathogenesis is facilitated by a privileged inflammatory microenvironment, with T regulatory FoxP3^+^ expressing T cells (Tregs) being a significant factor. PreImplantation Factor (PIF) is a peptide essential for pregnancy recognition and development. An immune modulatory function of the synthetic PIF analog (sPIF) has been successfully confirmed in multiple animal models. We report that PIF is expressed in the epithelial ectopic cells in close proximity to FoxP3^+^ stromal cells. We provide evidence that PIF interacts with FoxP3^+^ cells and modulates cell viability, dependent on cell source and presence of inflammatory mediators. Our finding represent a novel PIF-based mechanism in endometriosis that has potential for novel therapeutics.

## Introduction

Endometriosis is a chronic, benign disease affecting 10% of women in their reproductive years and characterized by the presence of stromal and epithelial cells outside the uterine cavity [1]. Endometriosis lesions are found predominantly in the pelvis, potentially through the reflux of viable endometrial cells during retrograde menstruation [1, 2]. However, recent evidence suggests that the pathogenesis of endometriosis requires more than retrograde menstruation, underlying the emerging role of stems cells and the immune response [3–5]. For example, the cyclic regeneration of a healthy endometrium depends on stem cells. Both endometrial- and bone marrow-derived stem cells may migrate to ectopic lesions and contribute to lesion growth [3]. Irrespective of their source however, once endometrial cells are present they secrete chemokines that stimulate immune cell infiltration [6]. Both the endometrial and infiltrating immune cells produce inflammatory cytokines, such as TNFα, that further stimulate a cascading inflammatory response. This creates a regulatory feed forward loop influencing both the progression and symptomology of the disease [7, 8], resulting, in a unique microenvironment that contributes to these lesions being able to evade immune surveillance [4].

One of the key regulators of immune processes in endometriosis are regulatory T cells (Tregs) derived from CD4 lineage [9]. Tregs are produced naturally in the thymus and express the forkhead box P3 transcription factor (Foxp3^+^). Cytokine-induced increase of Foxp3 expression drives Tregs differentiation/activation and suppresses the response of effector T cells by inhibiting dendritic, or the other antigen presenting cells from triggering effector T cell proliferation [10]. Additionally, Tregs induce immune tolerance by production of IL-10, Transforming Growth Factor-β, and anti-inflammatory cytokines that inhibit T helper cell activation. Not surprisingly, this altered immunological microenvironment may lead to tumor growth [11–13]. On the contrary, the absence or depletion of Tregs lead to multi-systemic autoimmunity in mice and humans [14]. In line with a role for a modified immunity in the pathogenesis of endometriosis, CD4^+^/FoxP3^+^ Tregs are present in endometriotic lesions [15, 16].

PreImplantation Factor (PIF) is a fifteen amino acid linear peptide secreted by viable embryos [17, 18] that targets immune cells directly [19, 20]. PIF`s essential roles in pregnancy begins by priming the endometrium for implantation and continue by promoting trophoblast invasion through local and systemic immune modulation [21–25]. Not surprisingly, a synthetic analog of PIF (sPIF) was successfully tested in multiple non-pregnant and pregnant animal models including autoimmune, transplantation, radiation induced, and brain diseases [26–33]. For example, sPIF is neuroprotective in neonatal rats through modulation of microRNA let-7 and Protein Kinase A and Protein Kinase C signalling in neuronal and microglial cells [29, 30]. The neuroprotective and immune modulatory effect was also observed in adult multiple sclerosis models [27, 32] and the regulation of immune function was observed in graft-versus-host, inflammatory induced fetal loss, autoimmune diabetes, and radiation induced injury models [26, 28, 31, 33]. Furthermore, sPIF is currently being tested in an ongoing University-sponsored, FDA Fast-Track approved clinical trial for an autoimmune disease manifested in liver inflammation (NCT02239562). Given that PIF is an essential and safe pregnancy product and pregnancy itself suppresses endometriosis, we aimed to evaluate the role of PIF within the endometriotic environment.

## Material and methods

### Production and labelling of synthetic PIF

Synthetic PIF_15_ (MVRIKPGSANKPSDD) and a scrambled peptide sequence with the same amino acids in random order (GRVDPSNKSMPKDIA) were synthesized by solid-phase peptide synthesis (Peptide Synthesizer, Applied Biosystems) employing Fmoc (9-fluorenylmethoxycarbonyl) chemistry at Bio-Synthesis, Inc. (Lewisville, TX, USA). Final purification was carried out by reversed-phase HPLC and the peptide identity verified by matrix-assisted laser desorption/ionization time-of-flight mass spectrometry and amino acid analysis at >95% purity. Fluorescein labeled FITC-PIF and scrambled PIF (FITC- PIFscr) were generated as previously reported [22, 34] and the anti-PIF monoclonal antibody against MVRIKPGSANKPSDD was generated in (Genway, SanDiego, CA, USA).

### Immunohistochemistry

Tissue specimens were obtained from 25 women who underwent laparoscopic surgery for severe endometriosis according to the revised criteria of the American Society Reproductive Medicine [35] (see S1 Table for details). The surgical procedures were carried out in CERM-Hungaria Institute, Rome, Italy, from September 2014 through April 2015. The study was approved by Ethical Committee of CERM-Hungaria (Istitutional Review Board approved 10-02-2014) and conducted according to the Helsinky’s Declaration of Human Rights. Each patient undergoing surgical procedures signed a written informed consent. Samples were obtained from the ectopic endometrium, ovarian endometriomas and peritoneal implants. A total of 25 eutopic endometria, 25 ovarian endometriomas and 10 peritoneal implants were collected from patients. Furthermore, the endometria of 10 healthy women were used as controls. Biopsy samples were fixed in 4% neutral buffered formalin overnight with subsequently paraffin embedding.

Before performing immunohistochemistry (IHC) tissue sections were stained with eosin and hematoxylin to select ectopic tissue containing epithelial cells. Serial sections of 5μm thick were used for IHC. Commercially available monoclonal antibodies were used for the detection of PIF (BioIncept, LLC, NJ, USA) and Foxp3 (number: sc-53876, Santa Cruz, CA, USA). IHC was performed according to manufacturer’s instructions. Briefly, tissue sections were dewaxed and re-hydrated and endogenous peroxidase activity quenched by incubation with 0.3% hydrogen peroxide in methanol for 30 minutes at room temperature. Sections were exposed to a non-immune block with normal horse serum for 30 minutes at room temperature. Incubation with the first antibody was carried out at 4° C overnight with a dilution of 1 to 100 for the monoclonal mouse anti-human PIF and with a dilution of 1 to 50 for the monoclonal mouse anti-human Foxp3. Thereafter tissue sections were labelled with the avidin-biotin-peroxidase detection system Vectastain (Vector Laboratories, Burlingthon VT, USA). Each step was followed by washing with PBS. Finally, 3,3'-diaminobenzidine (DAB) and/or 3-amino-9-ethylcarbazole (AEC) were used as chromogens for single or double staining. Counterstaining was performed with hematoxylin. The positive controls were previously confirmed PIF and Foxp3 positive tissue. Negative controls were performed by replacing the primary antibody with mouse immunoglobulin at the same concentration used previously.

A semi-quantitative statistical analysis of specific staining was performed using an HSCORE system [36], calculated using the following equation: HSCORE = SPi(I+1), where i is the intensity of staining with a value of 1, 2, or 3 (weak, strong or very strong respectively) and Pi is the percentage of stained cells at each intensity, varying from 0% to 100%. For all samples, ten microscopic fields were counted by two observers blinded to group status and three slides of each sample were analysed. The HSCORE analysis was performed separately for epithelial and stromal compartment (each observer performed 4 different HSCOREs for each slide). The intra-observer and inter-observer coefficient of variation were 3.4% and 4.2% respectively.

### FITC-PIF flow cytometry

We reported on FITC-PIF binding in both pregnant and non-pregnant patients previously [21]. Briefly, at Millenova Immunology Laboratories non-pregnant, infertile and first trimester pregnant patients undergoing fertility treatment signed a standard informed consent (CARI, Institute, Chicago, USA). All experiments were performed in accordance with the guidelines and regulations of CARI, Institute, Chicago and with the approval from the Institutional Review Board of the University of Illinois, Chicago in March 2006, Dr. R. Roussev, PI. Blood was sampled as part of their work-up process and excess sample used. To test whether PIF targets regulatory T-cells we used specific anti-CD4^+^, anti-CD25^+^, and anti-FoxP3^+^ antibodies (BD, Pharmingen, USA). Binding was compared with scrambled-FITC-PIF used as a negative control. We isolated peripheral blood mononuclear cells (PBMC) following separation using Ficoll-Hypaque, Binding to Isotype control served as negative controls. Two- three color staining was performed using conventional techniques. Fluorescence measurements (20,000–50,000 gated events per sample) were performed in a Coulter^®^ Epics^®^ XL™ Flow Cytometer using System II software for data acquisition and analysis (Beckman Coulter, Inc., Miami, FL, USA).

### Isolation and culture of in vitro cell models

After ethical approval was obtained from the Bern Cantonal Ethical Committee (KEK) and written informed consent provided by participants endometrial biopsies were collected via soft curette (Pipelle de Cornier, Laboratorie CCD, France) and stored in RNAlater at -80°C from women undergoing laparoscopic surgery at the University Hospital Bern, Switzerland, as described previously [37]. The pelvic cavity was examined and any endometriotic lesions were removed and the presence of endometriosis confirmed via histological investigation. Endometrial biopsies were collected from women with (n = 4) and without (n = 5) endometriosis. All surgeries were performed during the proliferative phase of the menstrual cycle. Primary endometrial stromal cells (ESC) from women with and without endometriosis were prepared via collagenase digestion and size exclusion protocols as described previously [38]. Isolated ESC were maintained in Iscoves’s modified Eagle medium (IMEM) (Invitrogen) supplemented with 10% fetal calf serum (fcs) (Invitrogen) and 1% antibiotic/antimycotic (Invitrogen). Immortalized epithelial cell lines were kindly provided by Professor Kyo, Kanazawa, Japan and were isolated from eutopic endometrium, EM E6/E7 [39] and an ectopic endometriomas, EM’osis, [40]. Epithelial cells were cultured in Dulbecco’s modified Eagles medium (DMEM) (invitrogen) with 10% fcs and 1% antibiotic/antimycotic.

### Analysis of sPIF influence on cell viability

To determine cell viability the CellTiter96 AQueous One Solution Cell Proliferation Assay (MTS) (Promega) was used. Cells were plated into 96 well plates at a density of approximately 6 x 10^3^/well. Sixteen hours prior to treatments cells were changed into a reduced serum media (0.5% FCS). sPIF was prepared by diluting into phosphate buffered saline (PBS) at a final concentration of 300nM. Subsequent 1:3 serial dilutions (111nM, 37nM, 12.3nM and 4.12nM) were prepared for a dose-response assay. Treatment was performed for a total of 48 hours with the treatment media replaced after the first 24 hours. Cell viability was measured via the addition of the tetrazolium compound provided with the kit and colorimetric change measured by spectrophotometer as per manufacturer’s instructions. A control (without sPIF) was included for each experiment and all subsequent values expressed as a percent of control. For assay including TNFα recombinant human TNFα (R&D, Cat No; 210-TA, Minneapolis, MN, USA) was diluted into PBS at 100ng/ml and included in treatment media.

### Whole transcriptome expression array

For whole transcriptome expression array analysis ectopic endometrioma (EM’osis) cells were seeded into 6 well plates at approximately 2x 10^5^/well and grown until approximately 80% confluent. Sixteen hours prior to treatment the cells were transferred to 0.5% FCS media to synchronize cell cycles. Treatment with 100nM PIF was performed for 48 hours with the treatment media replenished after 24 hours. After treatment period cells were lysed in Qiazol lysis buffer (Qiagen) and RNA isolated using the RNAeasy mini kit (Qiagen), as per the manufacturers instructions. RNA quantity was measured via the Nanodrop 2000 (Witec) and quality via the Bioanalyser 2000 (Agilent). RNA was considered of sufficient quality if RNA integrity number (RIN) was above 9.8. A final concentration for all samples was approximately 200ng/ml. 6 RNA samples were analysed using the Affymetrix platform according to the manual`s instructions (GeneChip® Human Transcriptome Array 2.0 and miRNA Array, Affymetrix).

### Statistical analysis

IHC data are reported as a mean ± standard deviation. Statistical analysis was performed by SPSS statistical package (Chicago, IL USA), using the Mann-Whitney Sum Rank test as appropriate. Analysis of cell viability in the *in vitro* cell models in response to PIF treatment was performed with a two-way analysis of variance (ANOVA) test with a post-hoc Sidak’s multiple comparison test to determine if cell viability was increased by treatment conditions from control. Significance was considered a value for p < 0.05 and analysis performed with Graphpad Prism 6. The raw microarray data was background-corrected, normalized using the RMA method as implemented in the R/Bioconductor package affy [41]. Probe sets where redefined using the alternative chip definition file [42]. Differential gene expression was calculated using the moderated t-test as described previously and implemented in the R/Bioconductor package limma [43].

For Pathway analysis the output of limma was used to perform gene set enrichment analysis (GSEA) using the SetRank method. The key principle of this algorithm is that it discards gene sets that have initially been flagged as significant, if their significance is only due to the overlap with another gene set. It calculates the p-value of a gene set using the ranking of its genes in the ordered list of p-values as calculated by limma. The following databases were searched for significant gene sets: BIOCYC, Gene Ontology, KEGG, Pathway Interaction Database, REACTOME, and WikiPathways.

## Results

### PIF imparts epithelial ectopic endometria

**Fig 1:**
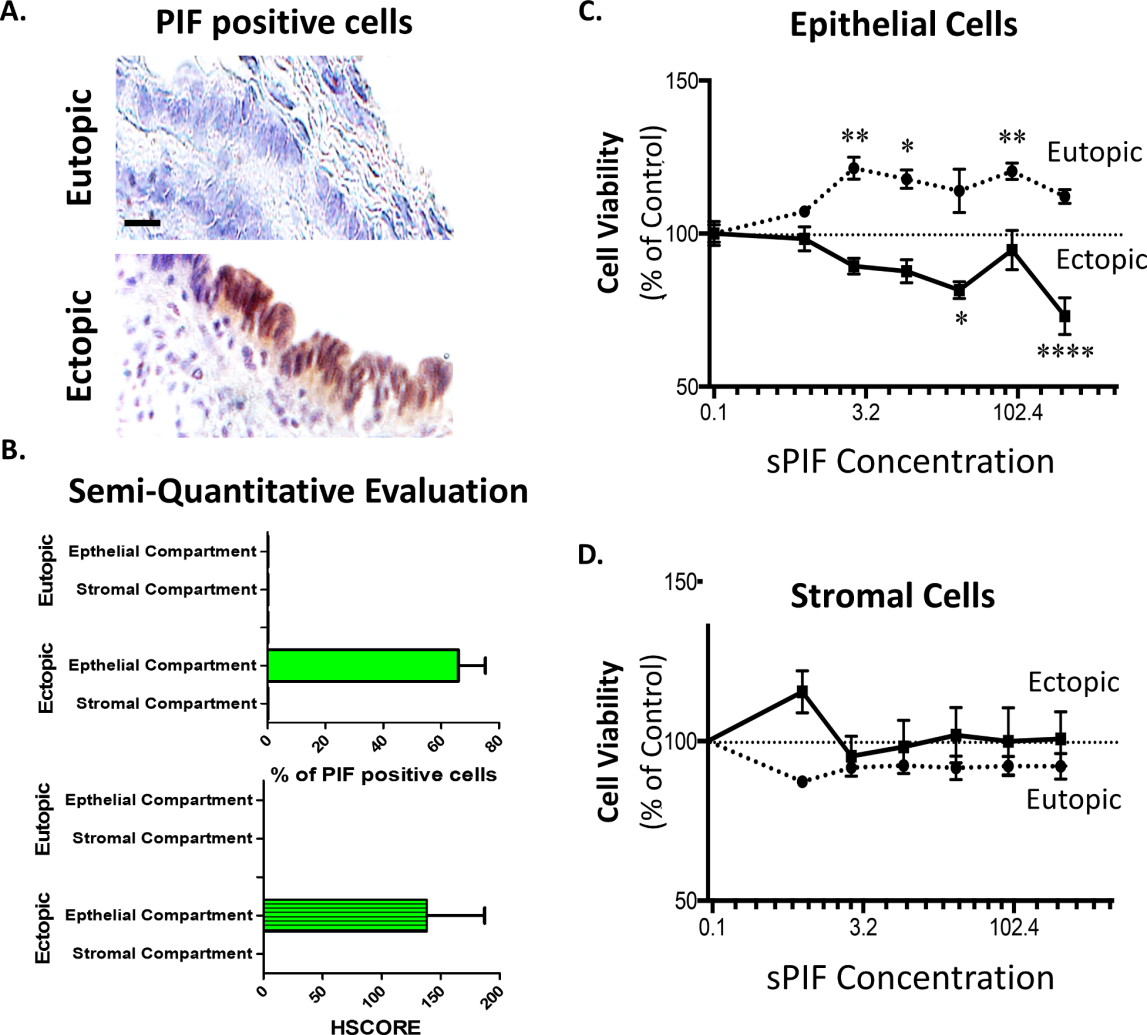
PIF re-expresses in epithelial ectopic cells and modulates cell viability. **(A)** PIF positive cells were not detected in tissues derived from healthy patients (upper panel). PIF positive cells were detected in epithelial compartment of ectopic endometrial tissues (lower panel). (**B)** Semi-quantitative analysis of PIF staining. We detected increased number of PIF positive cells with high HSCORE in epithelial compartment of ectopic tissues only. (**C) and (D)** Diverse influence of sPIF on cell survival. (**C)** sPIF treatment of epithelial ectopic cells (EM ‘osis −) resulted in a significant decrease of cell viability. However, treatment of epithelial eutopic cells (EM E6/E7 --) resulted in a significant increase of cell viability. (**D)** The viability of ectopic (−) and eutopic (--) stromal cells did not show a significant change in response to sPIF treatment. *p<0.05; **; p<0.01; ***p<0.001. Scale bar 20 μm.

To determine the role of PIF in endometriosis, tissue samples were obtained from women with or without endometriosis including the ectopic ovarian endometriomas and peritoneal implants during the proliferative and secretory phases (see S1 Table for characterization of the patients). We did not detect PIF positive cells in either the epithelial, or stromal cells of healthy controls in the proliferative and secretory phases (Fig 1A and 1B). However, we detected PIF positive cells in the epithelial cells of ectopic endometria, predominantly in the secretory phase documenting for the first time expression of PIF outside of pregnancy. We hypothesized that PIF expression may be induced as a protective mechanism so we tested the effect of sPIF using an *in-vitro* system.

To elucidate the potential role of PIF in endometriosis we used epithelial cell lines isolated from the eutopic endometrium (EM E6/E7) [39] and ectopic endometriomas (EM’osis) [40] and primary endometrial stromal cells (ESC) from women with and without endometriosis. We tested sPIF treatment in ascending doses in epithelial cells first. sPIF treatment significantly decreased cell viability compared to control (Fig 1C solid line). Furthermore, and supporting PIF`s role during embryo implantation, sPIF also increased the viability of eutopic endometrium cells (Fig 1D dashed line) [20]. In line with our previous reports and observation in human tissues (Fig 1A), sPIF treatment did not alter stromal cell viability (Fig 1D) [22]. Together, both PIF expression and sPIF treatment influence epithelial, but not stromal endometriotic cells suggesting a novel pathogenesis of endometriosis. To further dissect the underlying mechanisms, we performed a global gene expression array from sPIF treated ectopic endometrial epithelial cells.

### sPIF modulates T-cell receptor signaling

**Fig 2:**
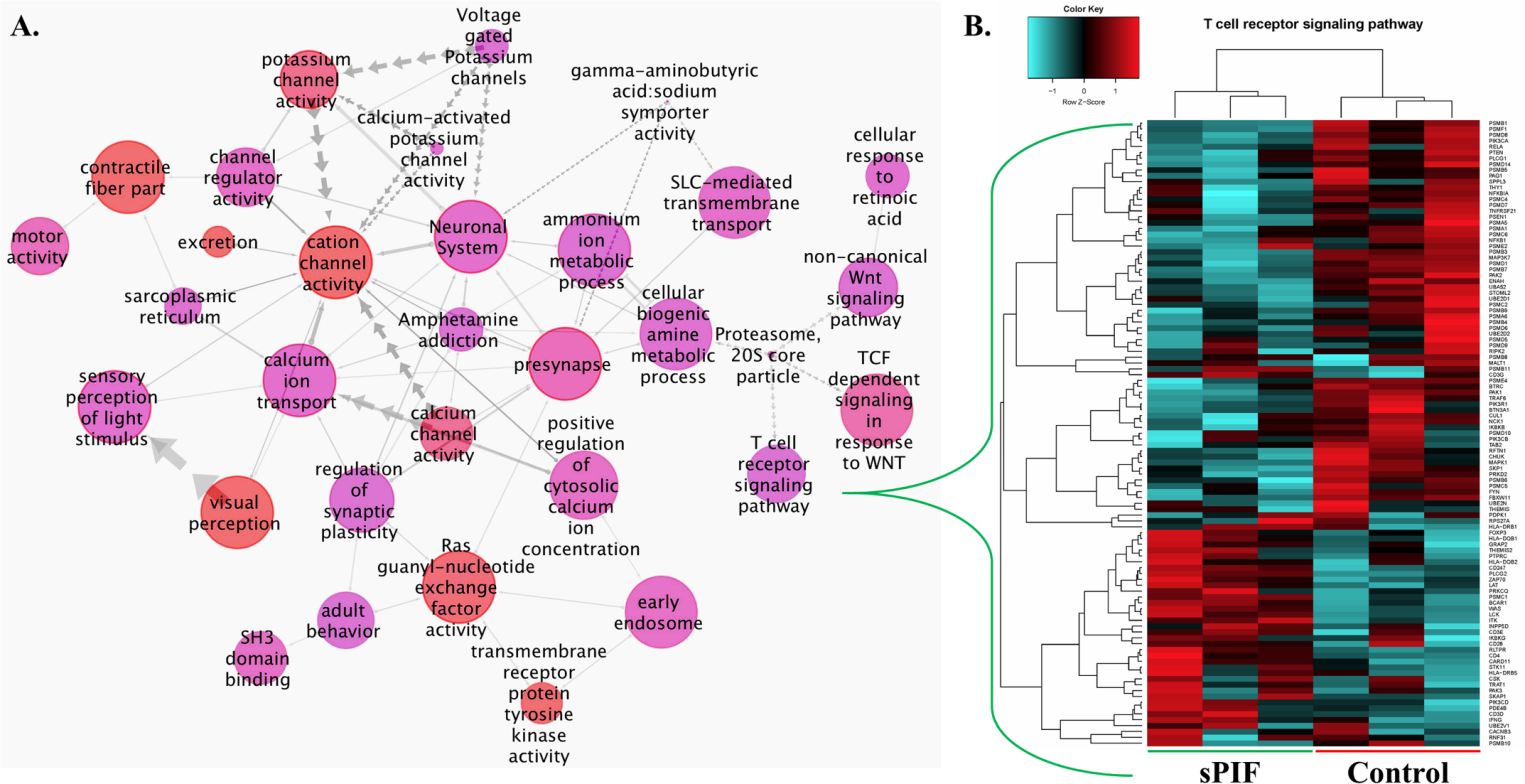
sPIF treatment results in global gene changes including T cell receptor signalling. **(A)** Gene set network showing the relations between the significant gene sets after sPIF treatment of epithelial ectopic cells. Every node represents a gene set and every edge reflects the intersection between two sets. Node size reflects the number of genes in each gene set; node fill color reflects the p-value corrected for intersections with the color going from blue to red with decreasing p-value; node border reflects network prominence, how important a particular gene set is compared to others. Edge width reflects the relative size of the intersection between two gene sets. Edge direction, as indicated by the arrow heads, is towards to more significant gene set. Repeated arrows indicate subset relations. (**B)** Detailed analysis of the T cell receptor signaling pathways showing multiple up (red) and down (blue) regulated genes (6 samples: 3 sPIF treated and 3 control samples).

Having a screening approach in mind, we performed a global gene array from sPIF`s treated epithelial ectopic cell lines (Fig 1). In line with previous reports [23], sPIF treatment resulted in modulation of multiple signaling pathways as shown by string analysis (see Fig 2A and S2 Table for details). We detected significant changes in channels activity pathways with cation and potassium channel activity being crucial, suggestion they play a crucial role in sPIF function, especially as potassium channels have previously been identified as a sPIF target [44]. Multiple pathways involved in neuronal development, plasticity, and protection were influenced by PIF treatment, in line with previous reports of sPIF neuroprotective effects [29, 30, 45]. Interestingly, changes in T-cell receptor signalling were also detected (see Fig 2A green arrow and S3 Table for details) and T cell signalling has previously been related to endometriosis pathogenesis [5, 46]. Therefore, we performed a detailed heat map analysis and detected multiple genes modulated by sPIF treatment (Fig 2B). Of special interest is the *FoxP3* gene as FoxP3^+^ Tregs cells are not decreased in endometriotic lesions during the secretory phase, which may contribute to their survival [5]. Given the importance of FoxP3 in the pathogenesis of endometriosis [5, 16], we aimed to confirm a potential PIF interaction with FoxP3 signaling.

### PIF interacts with FoxP3 positive cells

**Fig 3:**
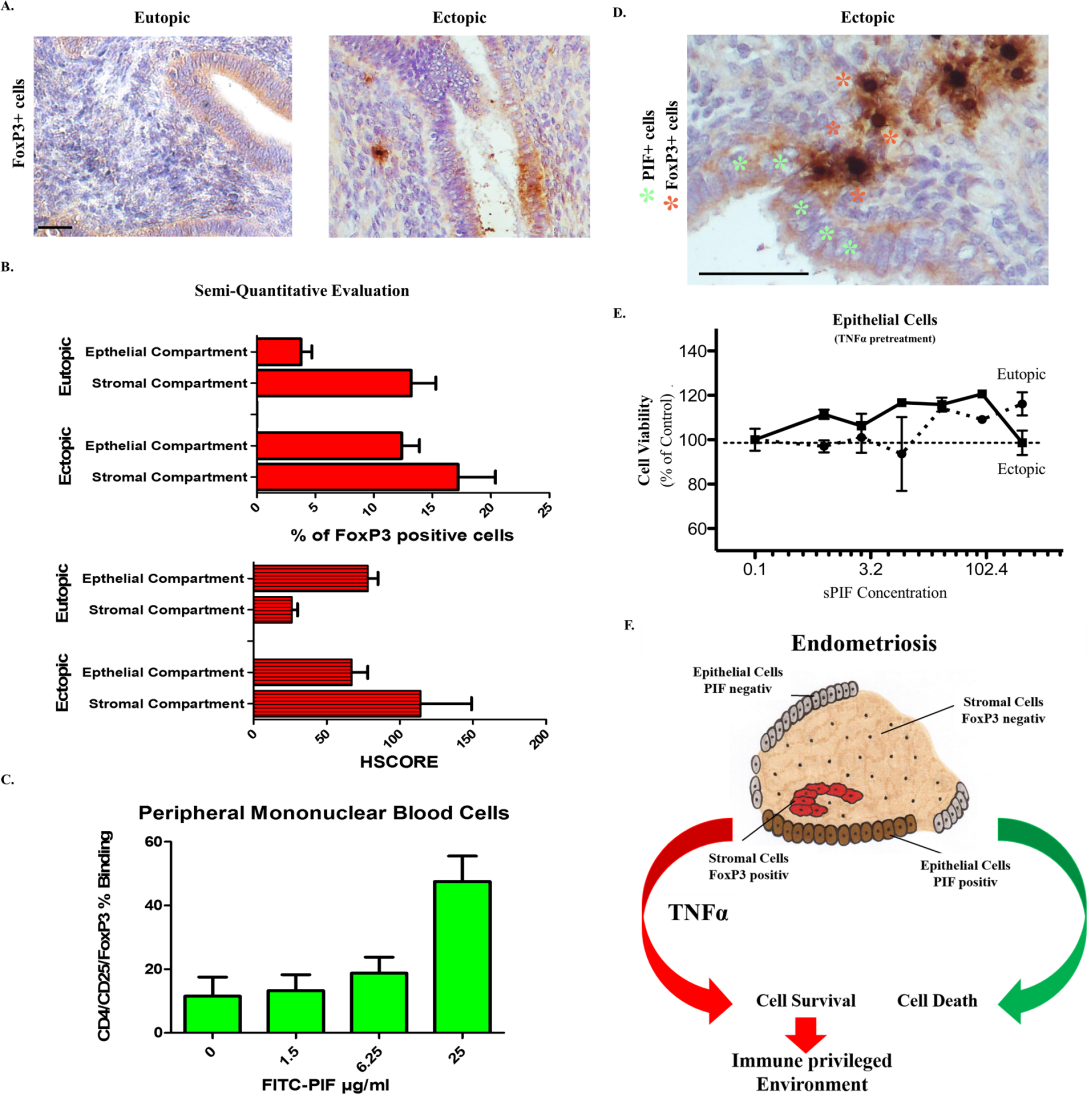
PIF interacts with FoxP3 positive cells. **(A)** FoxP3 positive cells were detected in both healthy (left panel) and endometriotic (right panel) tissues in stromal and epithelial compartments. (**B)** Semi-quantitative analysis of FoxP3 staining demonstrates increased percentage of positive cells with higher HSCORE in stromal compartment of ectopic tissues. (**C)** Dose dependent and specific PIF binding to Tregs. (**D)** Epithelial PIF positive cells (green asterisk) in close proximity to FoxP3 positive (red asterisk) stromal cells in ectopic tissue. (**E)** sPIF specific modulation of cell viability (Fig 1C) is abolished in the presence of TNFα. (**F)** New Hypothesis of immune privileged environment in endometriosis. PIF re-expression leads to recruitment of FoxP3 positive cells into the lesion. The production of pro-inflammatory cytokines such as TNFα leads to a diverged PIF effects resulting in cell survival. Systemic sPIF application may lead to absents of TNFα production and FoxP3 recruitment leading to cell death due to PIF re-expression in the endometriotic lesions. Scale bar 20 μm.

FoxP3^+^ cells were detected in both eutopic and ectopic tissues (Fig 3A and 3B) and in line with previous reports, we detected an increased number of FoxP3^+^ cells in the ectopic endometria, particularly during the secretory phase [15, 16]. To further underline the potential interaction between PIF and FoxP3^+^ cells, we tested PIF`s effects in a well-defined system of peripheral non-pregnant blood mononuclear cells (PBMCs). Notably, we reported on FITC-PIF binding to CD14^+^ and CD3^+^ cells in both pregnant and non-pregnant patients previously [21]. In addition binding to activated CD4^+^ cells were documented. Using the same method of flow cytometry, we detected a dose dependent increase in the binding of FITC-PIF to CD4^+^/CD25^+^/FoxP3^+^ cells (Fig 3C). This observation is in line with close proximity of FoxP3^+^ cells (stromal compartment) and PIF^+^ cells (epithelial compartment) of the ectopic endometria during the secretory phase (Fig 3D). Together, these observations suggest that the expression of PIF may contribute to the increased FoxP3 expression in these lesions.

Finally, we aimed to detect sPIF effects in the inflammatory endometriotic environment. Notably, multiple chemokines affect FoxP3^+^ cell function and FoxP3 cells regulate the survival of ectopic endometrial cells [9]. We decided to test sPIF effect on cell viability in the presence of TNFα as TNFα was identified as one of the pivotal chemokines involved in endometriosis and cell death [4]. Indeed, in the presence of TNFα, sPIF`s specific effect on cell viability in epithelial ectopic cells (Fig 1C) was abolished suggesting the pivotal involvement of TNFα (Fig 3E) in sPIF`s mediated effects. This observation supports the notion of a disturbed balance between pro-inflammatory and anti-inflammatory factors in endometriosis affecting the cellular survival [47].

Combining this data together therefore, we hypothesize that PIF expresses in the epithelial compartment of ectopic endometria resulting in the recruitment of FoxP3^+^ cells into the stromal compartment. The additional influx of chemokines and pro-inflammatory factors such as TNFα result in divergent sPIF effect on these cells creating a positive feed-back loop. It may lead to cellular survival which again contribute to a significant control of the immune privilege environment in the peritoneal cavity (Fig 3F). Thus, we hypothesize that PIF may be a crucial factor contributing to the pathogenesis of endometriosis on the local level. However, the immune modulatory effect of sPIF applied peripherally as a therapeutic option may lead to a decreased inflammatory response on the global level, leading to the recovery of proper immune balance on the local level and finally cell death of ectopic endometria. Studies investigating this hypothesis are currently underway.

## Discussion

Our study shows for the first time the presence of FoxP3^+^ cells in the endometriotic lesion in close proximity to PIF expressing epithelial cells (Fig 3D). The presence of Tregs in eutopic and ectopic endometrium of women with endometriosis has been reported previously [15, 16]. *FoxP3* mRNA is increased in ectopic endothelial tissue and the percentage of Tregs is significantly decreased in the peripheral blood of women with endometriosis, compared to healthy controls. However in the peritoneal fluid the Treg percentage is increased [48]. This discrepancy suggests a differential immune modulatory system at the local and global levels. Given that sPIF influences ectopic endometrial cells and potentially interacts with FoxP3^+^ cells (Figs 1 and 3), we speculate that PIF expression may mediate an immune privilege for endometriotic lesions. A similar role was previously reported for the Fas-FasLigand system [49]. Notably, in autoimmune disorders CD4^+^/CD25^+^/FoxP3^+^ dependent suppression of effector cells (macrophages, natural killer, dendritic, and cytotoxic T cells) was reported [5, 46]. As FoxP3^+^ cells were present in ectopic and eutopic epithelial cells it is possible that FoxP3 contributes to epithelial transition and differentiation as a transcription factor rather than a reaction to the inflammatory process as in case of TNFα [4].

Recent evidence suggests apoptotic epithelial cells contribute to Treg survival and abundance [50]. A role for PIF in mediating this effect would be plausible as cellular expression increases close to implantation and thus is associated with the development of immune tolerance for the embryo [22, 24]. The observation that PIF may interact with those cells (Fig 3D) combined with their close localization (Figs 1 and 3D) support a potential regulatory function, as shown in both *in vitro* and *in vivo* setting in CD4^+^ cells. It also raises the possibility that a similar action takes place locally at the site of endometriosis. A number of preclinical models have documented that PIF has both a local and global protective effects [20] and a potential for a bi-directional communication between PIF expressing epithelial cells and Treg cells exists. PIF increased cell viability of epithelial cells derived from the eutopic endometrium and decreased cell viability of cells derived from ectopic tissue (Fig 1C and 1D). These results support a local paracrine effects of PIF on epithelial cells within the endometriotic lesion and this effect may be modulated by the inflammatory microenvironment via TNFα (Fig 3E and 3F). TNFα is a hierarchal cytokine that stimulates the expression of numerous other inflammatory mediators though the activation of the IKKβ/NFκB pathway, a pathway specifically activated in the epithelial cells of endometriotic lesions [51]. The whole genome transcriptome analysis (Fig 2) showed a significant influence on the expression of T cell receptor signalling pathways raising the possibility that not only does the paracrine effects of PIF influence the survival of ectopic epithelial cells, but that it also influences gene expression and the ability of these epithelial cells to respond to the immune regulating effects of infiltrating Tregs.

The variation in response to PIF between the eutopic and ectopic derived epithelial cells is also of interest (Figs 1 and 3). Although the pathogenesis of endometriosis is still not resolved, the notion that ectopic endometriotic cells have an inherent characteristic that leads to implantation is intriguing [3]. Such as a pathological alteration may be due to the ectopic environment or may have the origin in stem cells [3]. Since embryo development is largely dependent on PIF and the fact that sPIF promotes cultured embryo and stem cell development support such a premise [17, 31, 52]. Although hypothetical the idea that a peptide such as PIF may be the missing link between the role of stems cells and immune responses in the pathogenesis of endometriosis is intriguing [3–5]. Further investigation is needed. Lastly, endometriotic derived PIF may also influence the symptomology of endometriosis. sPIF is a well described neuroprotective compound [20]. Recent evidence suggest that the pain produced by endometriosis occurs through an interaction with endometriotic associated nerve fibers detected in close proximity to the lesions [53, 54]. The neuroprotective effects of epithelia derived PIF may stimulate an enhanced nerve presence and influence the interpretation of pain in endometriosis. On the other hand peripheral sPIF and the ensuing reduced neuroinflammation may limit such neurotropic pain as well [30, 45].

## Supporting information

S1 Table Clinical and histopathological characteristics of patients included in the study. (DOCX)

S2 Table Global gene analysis. (XLSX)

S3 Table Detailed analysis of T-Cell receptor signaling. (XML)

## References

[1] Giudice LC. Clinical practice. Endometriosis. N Engl J Med. 2010;362(25):2389–98. doi: 10.1056/NEJMcp1000274 ; PubMed Central PMCID: PMCPMC3108065. 20573927 10.1056/NEJMcp1000274 PMC3108065

[2] Sampson JA. Metastatic or Embolic Endometriosis, due to the Menstrual Dissemination of Endometrial Tissue into the Venous Circulation. Am J Pathol. 1927;3(2):93–110 43. ; PubMed Central PMCID: PMCPMC1931779. 19969738 PMC1931779

[3] Macer ML, Taylor HS. Endometriosis and infertility: a review of the pathogenesis and treatment of endometriosis-associated infertility. Obstet Gynecol Clin North Am. 2012;39(4):535–49. doi: 10.1016/j.ogc.2012.10.002 ; PubMed Central PMCID: PMCPMC3538128. 23182559 10.1016/j.ogc.2012.10.002 PMC3538128

[4] Han SJ, Jung SY, Wu SP, Hawkins SM, Park MJ, Kyo S, et al Estrogen Receptor beta Modulates Apoptosis Complexes and the Inflammasome to Drive the Pathogenesis of Endometriosis. Cell. 2015;163(4):960–74. doi: 10.1016/j.cell.2015.10.034 ; PubMed Central PMCID: PMCPMC4640214. 26544941 10.1016/j.cell.2015.10.034 PMC4640214

[5] Berbic M, Fraser IS. Regulatory T cells and other leukocytes in the pathogenesis of endometriosis. J Reprod Immunol. 2011;88(2):149–55. doi: 10.1016/j.jri.2010.11.004 . 21269709 10.1016/j.jri.2010.11.004

[6] Hornung D, Ryan IP, Chao VA, Vigne JL, Schriock ED, Taylor RN. Immunolocalization and regulation of the chemokine RANTES in human endometrial and endometriosis tissues and cells. J Clin Endocrinol Metab. 1997;82(5):1621–8. doi: 10.1210/jcem.82.5.3919 . 9141560 10.1210/jcem.82.5.3919

[7] Bersinger NA, Frischknecht F, Taylor RN, Mueller MD. Basal and cytokine-stimulated production of epithelial neutrophil activating peptide-78 (ENA-78) and interleukin-8 (IL-8) by cultured human endometrial epithelial and stromal cells. Fertil Steril. 2008;89(5 Suppl):1530–6. doi: 10.1016/j.fertnstert.2007.05.075 . 17761179 10.1016/j.fertnstert.2007.05.075

[8] Bersinger NA, Gunthert AR, McKinnon B, Johann S, Mueller MD. Dose-response effect of interleukin (IL)-1beta, tumour necrosis factor (TNF)-alpha, and interferon-gamma on the in vitro production of epithelial neutrophil activating peptide-78 (ENA-78), IL-8, and IL-6 by human endometrial stromal cells. Arch Gynecol Obstet. 2011;283(6):1291–6. doi: 10.1007/s00404-010-1520-3 . 20505949 10.1007/s00404-010-1520-3

[9] Li MQ, Wang Y, Chang KK, Meng YH, Liu LB, Mei J, et al CD4+Foxp3+ regulatory T cell differentiation mediated by endometrial stromal cell-derived TECK promotes the growth and invasion of endometriotic lesions. Cell Death Dis. 2014;5:e1436 doi: 10.1038/cddis.2014.414 ; PubMed Central PMCID: PMCPMC4649519. 25275597 10.1038/cddis.2014.414 PMC4649519

[10] Tang Q, Bluestone JA. Regulatory T-cell physiology and application to treat autoimmunity. Immunol Rev. 2006;212:217–37. doi: 10.1111/j.0105-2896.2006.00421.x . 16903917 10.1111/j.0105-2896.2006.00421.x

[11] Bergmann C, Wild CA, Narwan M, Lotfi R, Lang S, Brandau S. Human tumor-induced and naturally occurring Treg cells differentially affect NK cells activated by either IL-2 or target cells. Eur J Immunol. 2011;41(12):3564–73. doi: 10.1002/eji.201141532 . 21905023 10.1002/eji.201141532

[12] Strauss L, Bergmann C, Gooding W, Johnson JT, Whiteside TL. The frequency and suppressor function of CD4+CD25highFoxp3+ T cells in the circulation of patients with squamous cell carcinoma of the head and neck. Clin Cancer Res. 2007;13(21):6301–11. doi: 10.1158/1078-0432.CCR-07-1403 . 17975141 10.1158/1078-0432.CCR-07-1403

[13] Wang W, Hodkinson P, McLaren F, MacKinnon A, Wallace W, Howie S, et al Small cell lung cancer tumour cells induce regulatory T lymphocytes, and patient survival correlates negatively with FOXP3+ cells in tumour infiltrate. Int J Cancer. 2012;131(6):E928–37. doi: 10.1002/ijc.27613 . 22532287 10.1002/ijc.27613

[14] Buckner JH. Mechanisms of impaired regulation by CD4(+)CD25(+)FOXP3(+) regulatory T cells in human autoimmune diseases. Nat Rev Immunol. 2010;10(12):849–59. doi: 10.1038/nri2889 ; PubMed Central PMCID: PMCPMC3046807. 21107346 10.1038/nri2889 PMC3046807

[15] Budiu RA, Diaconu I, Chrissluis R, Dricu A, Edwards RP, Vlad AM. A conditional mouse model for human MUC1-positive endometriosis shows the presence of anti-MUC1 antibodies and Foxp3+ regulatory T cells. Dis Model Mech. 2009;2(11–12):593–603. doi: 10.1242/dmm.002535 . 19841240 10.1242/dmm.002535

[16] Berbic M, Hey-Cunningham AJ, Ng C, Tokushige N, Ganewatta S, Markham R, et al The role of Foxp3+ regulatory T-cells in endometriosis: a potential controlling mechanism for a complex, chronic immunological condition. Hum Reprod. 2010;25(4):900–7. doi: 10.1093/humrep/deq020 . 20150173 10.1093/humrep/deq020

[17] Stamatkin CW, Roussev RG, Stout M, Absalon-Medina V, Ramu S, Goodman C, et al PreImplantation Factor (PIF) correlates with early mammalian embryo development-bovine and murine models. Reprod Biol Endocrinol. 2011;9:63 doi: 10.1186/1477-7827-9-63 ; PubMed Central PMCID: PMCPMC3112407. 21569635 10.1186/1477-7827-9-63 PMC3112407

[18] Ramu S, Stamatkin C, Timms L, Ruble M, Roussev RG, Barnea ER. PreImplantation factor (PIF) detection in maternal circulation in early pregnancy correlates with live birth (bovine model). Reprod Biol Endocrinol. 2013;11:105 doi: 10.1186/1477-7827-11-105 ; PubMed Central PMCID: PMCPMC3842769. 24238492 10.1186/1477-7827-11-105 PMC3842769

[19] Barnea ER, Hayrabedyan S, Todorova K, Almogi-Hazan O, Or R, Guingab J, et al PreImplantation factor (PIF*) regulates systemic immunity and targets protective regulatory and cytoskeleton proteins. Immunobiology. 2016;221(7):778–93. doi: 10.1016/j.imbio.2016.02.004 . 26944449 10.1016/j.imbio.2016.02.004

[20] Barnea ER, Almogi-Hazan O, Or R, Mueller M, Ria F, Weiss L, et al Immune regulatory and neuroprotective properties of preimplantation factor: From newborn to adult. Pharmacology & therapeutics. 2015;156:10–25. doi: 10.1016/j.pharmthera.2015.10.008 . 26546485 10.1016/j.pharmthera.2015.10.008

[21] Barnea ER, Kirk D, Ramu S, Rivnay B, Roussev R, Paidas MJ. PreImplantation Factor (PIF) orchestrates systemic antiinflammatory response by immune cells: effect on peripheral blood mononuclear cells. Am J Obstet Gynecol. 2012;207(4):313 e1–11. doi: 10.1016/j.ajog.2012.07.017 . 23021695 10.1016/j.ajog.2012.07.017

[22] Barnea ER, Kirk D, Paidas MJ. Preimplantation factor (PIF) promoting role in embryo implantation: increases endometrial integrin-alpha2beta3, amphiregulin and epiregulin while reducing betacellulin expression via MAPK in decidua. Reprod Biol Endocrinol. 2012;10:50 doi: 10.1186/1477-7827-10-50 ; PubMed Central PMCID: PMCPMC3444419. 22788113 10.1186/1477-7827-10-50 PMC3444419

[23] Paidas MJ, Krikun G, Huang SJ, Jones R, Romano M, Annunziato J, et al A genomic and proteomic investigation of the impact of preimplantation factor on human decidual cells. Am J Obstet Gynecol. 2010;202(5):459 e1–8. Epub 2010/05/11. doi: 10.1016/j.ajog.2010.03.024 ; PubMed Central PMCID: PMC2867836. 20452489 10.1016/j.ajog.2010.03.024 PMC2867836

[24] Duzyj CM, Barnea ER, Li M, Huang SJ, Krikun G, Paidas MJ. Preimplantation factor promotes first trimester trophoblast invasion. Am J Obstet Gynecol. 2010;203(4):402 e1–4. doi: 10.1016/j.ajog.2010.06.060 ; PubMed Central PMCID: PMCPMC2947608. 20708167 10.1016/j.ajog.2010.06.060 PMC2947608

[25] Moindjie H, Santos ED, Loeuillet L, Gronier H, de Mazancourt P, Barnea ER, et al Preimplantation factor (PIF) promotes human trophoblast invasion. Biol Reprod. 2014;91(5):118 doi: 10.1095/biolreprod.114.119156 . 25232018 10.1095/biolreprod.114.119156

[26] Weiss L, Bernstein S, Jones R, Amunugama R, Krizman D, Jebailey L, et al Preimplantation factor (PIF) analog prevents type I diabetes mellitus (TIDM) development by preserving pancreatic function in NOD mice. Endocrine. 2011;40(1):41–54. doi: 10.1007/s12020-011-9438-5 . 21424847 10.1007/s12020-011-9438-5

[27] Weiss L, Or R, Jones RC, Amunugama R, JeBailey L, Ramu S, et al Preimplantation factor (PIF*) reverses neuroinflammation while promoting neural repair in EAE model. J Neurol Sci. 2012;312(1–2):146–57. doi: 10.1016/j.jns.2011.07.050 . 21996270 10.1016/j.jns.2011.07.050

[28] Azar Y, Shainer R, Almogi-Hazan O, Bringer R, Compton SR, Paidas MJ, et al Preimplantation factor reduces graft-versus-host disease by regulating immune response and lowering oxidative stress (murine model). Biol Blood Marrow Transplant. 2013;19(4):519–28. doi: 10.1016/j.bbmt.2012.12.011 . 23266739 10.1016/j.bbmt.2012.12.011

[29] Mueller M, Schoeberlein A, Zhou J, Joerger-Messerli M, Oppliger B, Reinhart U, et al PreImplantation Factor bolsters neuroprotection via modulating Protein Kinase A and Protein Kinase C signaling. Cell death and differentiation. 2015;22(12):2078–86. doi: 10.1038/cdd.2015.55 ; PubMed Central PMCID: PMCPMC4816111. 25976303 10.1038/cdd.2015.55 PMC4816111

[30] Mueller M, Zhou J, Yang L, Gao Y, Wu F, Schoeberlein A, et al PreImplantation factor promotes neuroprotection by targeting microRNA let-7. Proceedings of the National Academy of Sciences of the United States of America. 2014;111(38):13882–7. doi: 10.1073/pnas.1411674111 ; PubMed Central PMCID: PMCPMC4183321. 25205808 10.1073/pnas.1411674111 PMC4183321

[31] Shainer R, Almogi-Hazan O, Berger A, Hinden L, Mueller M, Brodie C, et al PreImplantation factor (PIF) therapy provides comprehensive protection against radiation induced pathologies. Oncotarget. 2016 doi: 10.18632/oncotarget.10635 . 27449294 10.18632/oncotarget.10635 PMC5312289

[32] Migliara G, Mueller M, Piermattei A, Brodie C, Paidas MJ, Barnea ER, et al PIF* promotes brain re-myelination locally while regulating systemic inflammation- clinically relevant multiple sclerosis M.smegmatis model. Oncotarget. 2017;8(13):21834–51. doi: 10.18632/oncotarget.15662 ; PubMed Central PMCID: PMCPMC5400627. 28423529 10.18632/oncotarget.15662 PMC5400627

[33] Di Simone N, Di Nicuolo F, Marana R, Castellani R, Ria F, Veglia M, et al Synthetic PreImplantation Factor (PIF) prevents fetal loss by modulating LPS induced inflammatory response. PLoS One. 2017;12(7):e0180642 doi: 10.1371/journal.pone.0180642 . 28704412 10.1371/journal.pone.0180642 PMC5507516

[34] Barnea ER, Kirk D, Ramu S, Rivnay B, Roussev R, Paidas MJ. PreImplantation Factor (PIF) orchestrates systemic antiinflammatory response by immune cells: effect on peripheral blood mononuclear cells. Am J Obstet Gynecol. 2012;207:313 doi: 10.1016/j.ajog.2012.07.017 23021695 10.1016/j.ajog.2012.07.017

[35] Revised American Society for Reproductive Medicine classification of endometriosis: 1996. Fertil Steril. 1997;67(5):817–21. . 9130884 10.1016/s0015-0282(97)81391-x

[36] McCarty KS Jr., Miller LS, Cox EB, Konrath J, McCarty KS Sr. Estrogen receptor analyses. Correlation of biochemical and immunohistochemical methods using monoclonal antireceptor antibodies. Arch Pathol Lab Med. 1985;109(8):716–21. . 3893381

[37] Santi A, Felser RS, Mueller MD, Wunder DM, McKinnon B, Bersinger NA. Increased endometrial placenta growth factor (PLGF) gene expression in women with successful implantation. Fertil Steril. 2011;96(3):663–8. doi: 10.1016/j.fertnstert.2011.06.039 . 21762891 10.1016/j.fertnstert.2011.06.039

[38] McKinnon BD, Evers J, Bersinger NA, Mueller MD. Induction of the neurokinin 1 receptor by TNFalpha in endometriotic tissue provides the potential for neurogenic control over endometriotic lesion growth. J Clin Endocrinol Metab. 2013;98(6):2469–77. doi: 10.1210/jc.2013-1019 . 23553861 10.1210/jc.2013-1019

[39] Kyo S, Nakamura M, Kiyono T, Maida Y, Kanaya T, Tanaka M, et al Successful immortalization of endometrial glandular cells with normal structural and functional characteristics. Am J Pathol. 2003;163(6):2259–69. doi: 10.1016/S0002-9440(10)63583-3 ; PubMed Central PMCID: PMCPMC1892381. 14633600 10.1016/S0002-9440(10)63583-3 PMC1892381

[40] Bono Y, Kyo S, Takakura M, Maida Y, Mizumoto Y, Nakamura M, et al Creation of immortalised epithelial cells from ovarian endometrioma. Br J Cancer. 2012;106(6):1205–13. doi: 10.1038/bjc.2012.26 ; PubMed Central PMCID: PMCPMC3304406. 22353808 10.1038/bjc.2012.26 PMC3304406

[41] Gautier L, Cope L, Bolstad BM, Irizarry RA. affy—analysis of Affymetrix GeneChip data at the probe level. Bioinformatics. 2004;20(3):307–15. doi: 10.1093/bioinformatics/btg405 . 14960456 10.1093/bioinformatics/btg405

[42] Dai M, Wang P, Boyd AD, Kostov G, Athey B, Jones EG, et al Evolving gene/transcript definitions significantly alter the interpretation of GeneChip data. Nucleic Acids Res. 2005;33(20):e175 doi: 10.1093/nar/gni179 ; PubMed Central PMCID: PMCPMC1283542. 16284200 10.1093/nar/gni179 PMC1283542

[43] Ritchie ME, Phipson B, Wu D, Hu Y, Law CW, Shi W, et al limma powers differential expression analyses for RNA-sequencing and microarray studies. Nucleic Acids Res. 2015;43(7):e47 doi: 10.1093/nar/gkv007 ; PubMed Central PMCID: PMCPMC4402510. 25605792 10.1093/nar/gkv007 PMC4402510

[44] Chen YC, Rivera J, Fitzgerald M, Hausding C, Ying YL, Wang X, et al PreImplantation factor prevents atherosclerosis via its immunomodulatory effects without affecting serum lipids. Thromb Haemost. 2016;115(5):1010–24. doi: 10.1160/TH15-08-0640 . 26842698 10.1160/TH15-08-0640

[45] Weiss L, Or R, Jones RC, Amunugama R, JeBailey L, Ramu S, et al Preimplantation factor (PIF*) reverses neuroinflammation while promoting neural repair in EAE model. J Neurol Sci. 2012;312(1–2):146–57. doi: 10.1016/j.jns.2011.07.050 . 21996270 10.1016/j.jns.2011.07.050

[46] Sakaguchi S, Yamaguchi T, Nomura T, Ono M. Regulatory T cells and immune tolerance. Cell. 2008;133(5):775–87. doi: 10.1016/j.cell.2008.05.009 . 18510923 10.1016/j.cell.2008.05.009

[47] Tagashira Y, Taniguchi F, Harada T, Ikeda A, Watanabe A, Terakawa N. Interleukin-10 attenuates TNF-alpha-induced interleukin-6 production in endometriotic stromal cells. Fertil Steril. 2009;91(5 Suppl):2185–92. doi: 10.1016/j.fertnstert.2008.04.052 . 18684450 10.1016/j.fertnstert.2008.04.052

[48] Olkowska-Truchanowicz J, Bocian K, Maksym RB, Bialoszewska A, Wlodarczyk D, Baranowski W, et al CD4(+) CD25(+) FOXP3(+) regulatory T cells in peripheral blood and peritoneal fluid of patients with endometriosis. Hum Reprod. 2013;28(1):119–24. doi: 10.1093/humrep/des346 . 23019301 10.1093/humrep/des346

[49] Sbracia M, Valeri C, Antonini G, Biagiotti G, Pacchiarotti A, Pacchiarotti A. Fas and Fas-Ligand in Eutopic and Ectopic Endometrium of Women With Endometriosis: The Possible Immune Privilege of Ectopic Endometrium. Reprod Sci. 2016;23(1):81–6. doi: 10.1177/1933719115594019 . 26156853 10.1177/1933719115594019

[50] Nakahashi-Oda C, Udayanga KG, Nakamura Y, Nakazawa Y, Totsuka N, Miki H, et al Apoptotic epithelial cells control the abundance of Treg cells at barrier surfaces. Nat Immunol. 2016;17(4):441–50. doi: 10.1038/ni.3345 . 26855029 10.1038/ni.3345

[51] Kocbek V, Grandi G, Blank F, Wotzkow C, Bersinger NA, Mueller MD, et al TNFalpha-induced IKKbeta complex activation influences epithelial, but not stromal cell survival in endometriosis. Mol Hum Reprod. 2016;22(11):768–77. doi: 10.1093/molehr/gaw054 . 27542948 10.1093/molehr/gaw054

[52] Stamatkin CW, Roussev RG, Stout M, Coulam CB, Triche E, Godke RA, et al Preimplantation factor negates embryo toxicity and promotes embryo development in culture. Reprod Biomed Online. 2011;23(4):517–24. doi: 10.1016/j.rbmo.2011.06.009 . 21900046 10.1016/j.rbmo.2011.06.009

[53] McKinnon BD, Bertschi D, Bersinger NA, Mueller MD. Inflammation and nerve fiber interaction in endometriotic pain. Trends Endocrinol Metab. 2015;26(1):1–10. doi: 10.1016/j.tem.2014.10.003 . 25465987 10.1016/j.tem.2014.10.003

[54] McKinnon B, Bersinger NA, Wotzkow C, Mueller MD. Endometriosis-associated nerve fibers, peritoneal fluid cytokine concentrations, and pain in endometriotic lesions from different locations. Fertil Steril. 2012;97(2):373–80. doi: 10.1016/j.fertnstert.2011.11.011 . 22154765 10.1016/j.fertnstert.2011.11.011

