# Supplementary material for: PreImplantation Factor in endometriosis: A potential role in inducing immune privilege for ectopic endometrium
Source: PLoS One. 2017 Sep 13;12(9):e0184399. doi: 10.1371/journal.pone.0184399 (PMC5597204; doi:10.1371/journal.pone.0184399)
Supplement: S1 Table — (DOCX) [file pone.0184399.s001.docx]

**PreImplantation Factor in endometriosis: a potential role in inducing immune privilege for ectopic endometrium**

Marco Sbracia, Brett McKinnon, Fabio Scarpellini, Daniela Marconi, Gabriele Rossi, Cedric Simmilion, Michael D Mueller, Eytan R. Barnea, Martin Mueller

**SUPPLEMENTARY MATERIAL**

**Supplementary Table 1.**

Clinical and histopathological characteristics of patients included in the study.

| **Endometriosis Patients n°** | **Age** | **Menstrual pase** | | **Anatomical phase** | **ASRM stage** |
| --- | --- | --- | --- | --- | --- |
| 1 | 31 | Proliferative | Eutopic,ovarian,peritoneal | | IV |
| 2 | 28 | Secretory | Eutopic,ovarian | | III |
| 3 | 32 | Proliferative | Eutopic,ovarian,peritoneal | | IV |
| 4 | 34 | Secretory | Eutopic,ovarian | | III |
| 5 | 36 | Proliferative | Eutopic,ovarian,peritoneal | | IV |
| 6 | 29 | Secretory | Eutopic,ovarian | | III |
| 7 | 38 | Proliferative | Eutopic,ovarian | | III |
| 8 | 40 | Secretory | Eutopic,ovarian | | IV |
| 9 | 30 | Proliferative | Eutopic,ovarian,peritoneal | | IV |
| 10 | 39 | Proliferative | Eutopic,ovarian | | III |
| 11 | 40 | Secretory | Eutopic,ovarian,peritoneal | | IV |
| 12 | 41 | Proliferative | Eutopic,ovarian,peritoneal | | IV |
| 13 | 37 | Secretory | Eutopic,ovarian,peritoneal | | IV |
| 14 | 34 | Secretory | Eutopic,ovarian,peritoneal | | IV |
| 15 | 29 | Proliferative | Eutopic,ovarian | | III |
| 16 | 30 | Secretory | Eutopic,ovarian | | III |
| 17 | 33 | Proliferative | Eutopic,ovarian | | IV |
| 18 | 35 | Secretory | Eutopic,ovarian,peritoneal | | IV |
| 19 | 34 | Proliferative | Eutopic,ovarian,peritoneal | | IV |
| 20 | 39 | Secretory | Eutopic,ovarian | | III |
| 21 | 29 | Secretory | Eutopic ovarian | | IV |
| 22 | 31 | Proliferative | Eutopic ovarian | | III |
| 23 | 34 | Secretory | Eutopic ovarian | | IV |
| 24 | 28 | Proliferative | Eutopic ovarian | | III |
| 25 | 35 | Secretory | Eutopic ovarian | | IV |
| **Healthy Controls n°** |  |  |  | |  |
| 1 | 28 | Proliferative | Eutopic | | - |
| 2 | 36 | Secretory | Eutopic | | - |
| 3 | 37 | Proliferative | Eutopic | | - |
| 4 | 38 | Secretory | Eutopic | | - |
| 5 | 35 | Secretory | Eutopic | | - |
| 6 | 32 | Proliferative | Eutopic | | - |
| 7 | 36 | Secretory | Eutopic | | - |
| 8 | 37 | Proliferative | Eutopic | | - |
| 9 | 39 | Secretory | Eutopic | | - |
| 10 | 40 | Secretory | Eutopic | | - |
